# Encapsulation of Amikacin into Microparticles Based on Low-Molecular-Weight Poly(lactic acid) and Poly(lactic acid-*co*-polyethylene glycol)

**DOI:** 10.1021/acs.molpharmaceut.1c00193

**Published:** 2021-07-01

**Authors:** Marta Glinka, Katerina Filatova, Justyna Kucińska-Lipka, Eva Domincova Bergerova, Andrzej Wasik, Vladimir Sedlařík

**Affiliations:** † Department of Analytical Chemistry, Faculty of Chemistry, Gdańsk University of Technology, G. Narutowicza 11/12, Gdańsk 80-233, Poland; ‡ Centre of Polymer Systems, University Institute, Tomas Bata University in Zlín, Tomáše Bati 5678, Zlín 76001, Czech Republic; § Department of Polymers Technology, Faculty of Chemistry, Gdańsk University of Technology, G. Narutowicza 11/12, Gdańsk 80-233, Poland

**Keywords:** poly(lactic acid), amikacin encapsulation, drug delivery systems, microparticles, targeted therapy

## Abstract

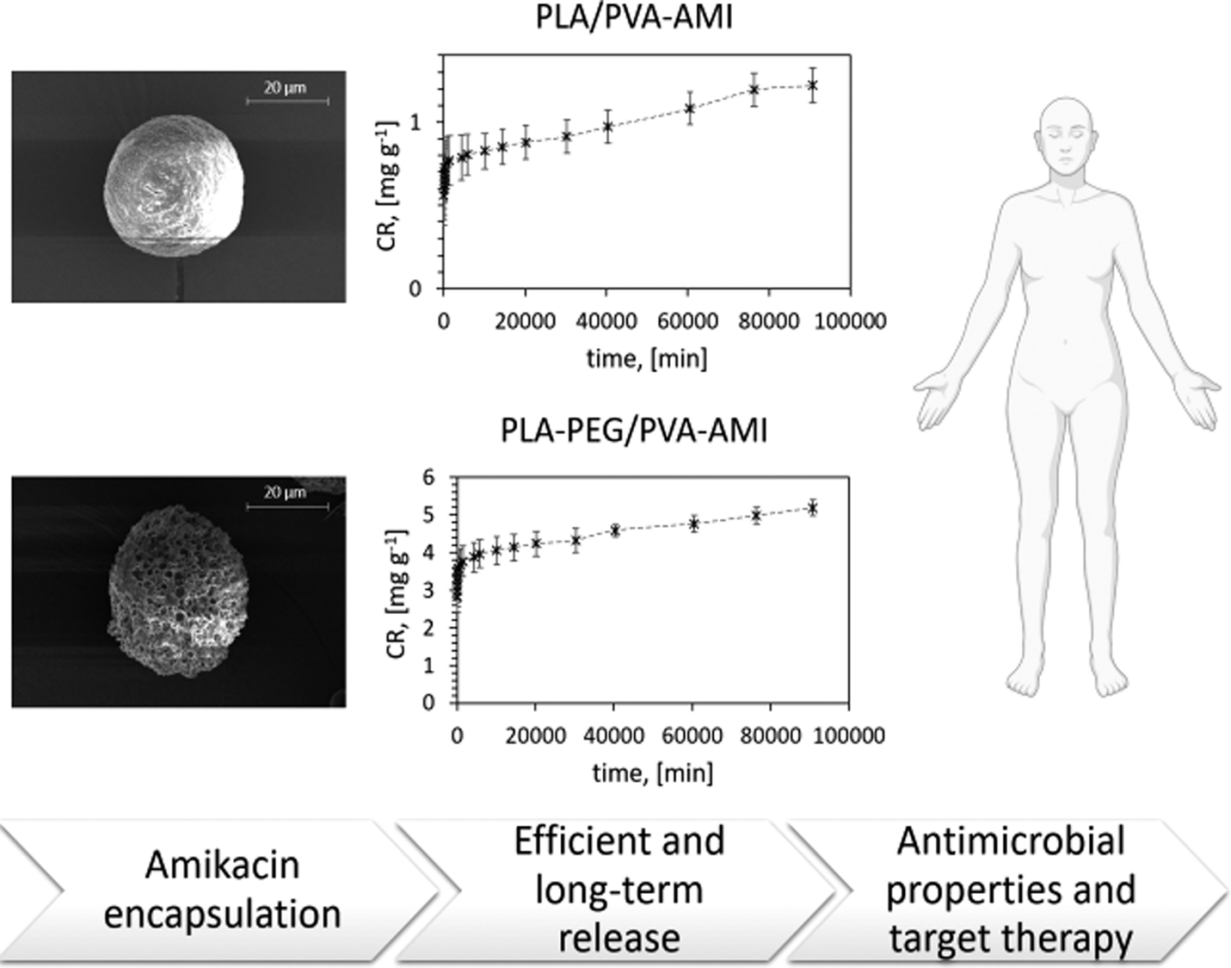

The aim of this study was to fabricate novel microparticles (MPs) for efficient and long-term delivery of amikacin (AMI). The emulsification method proposed for encapsulating AMI employed low-molecular-weight poly(lactic acid) (PLA) and poly(lactic acid-*co*-polyethylene glycol) (PLA–PEG), both supplemented with poly(vinyl alcohol) (PVA). The diameters of the particles obtained were determined as less than 30 μm. Based on an in-vitro release study, it was proven that the MPs (both PLA/PVA- and PLA–PEG/PVA-based) demonstrated long-term AMI release (2 months), the kinetics of which adhered to the Korsmeyer–Peppas model. The loading efficiencies of AMI in the study were determined at the followings levels: 36.5 ± 1.5 μg/mg for the PLA-based MPs and 106 ± 32 μg/mg for the PLA–PEG-based MPs. These values were relatively high and draw parallels with studies published on the encapsulation of aminoglycosides. The MPs provided antimicrobial action against the *Staphylococcus aureus*, *Escherichia coli*, *Pseudomonas aeruginosa*, and *Klebsiella pneumoniae* bacterial strains. The materials were also comprehensively characterized by the following methods: differential scanning calorimetry; gel permeation chromatography; scanning electron microscopy; Fourier transform infrared spectroscopy–attenuated total reflectance; energy-dispersive X-ray fluorescence; and Brunauer–Emmett–Teller surface area analysis. The findings of this study contribute toward discerning new means for conducting targeted therapy with polar, broad spectrum antibiotics.

## 1 Introduction

Amikacin (AMI) is a semisynthetic, broad spectrum aminoglycoside antibiotic, primarily used against infections caused by Gram-negative bacteria. Examples include *Escherichia coli*, potentially resulting in diseases affecting the digestive and urinary systems, or *Klebsiella pneumoniae* and *Pseudomonas aeruginosa*, which trigger nosocomial infections. AMI is less commonly applied against infections precipitated by Gram-positive bacteria such as *Staphylococcus epidermidis* or *Staphylococcus aureus*, both constituting sources of post-hospital infections.^1^

Despite its therapeutic action, AMI is known to have toxic side effects, especially nephrotoxicity and ototoxicity due to its low therapeutic index. Another drawback relates to poor absorption if administered orally, as a consequence of the polycationic nature of the molecule. For these reasons, medicating with AMI has to be a strictly controlled process.^2,3^

**Figure 1:**
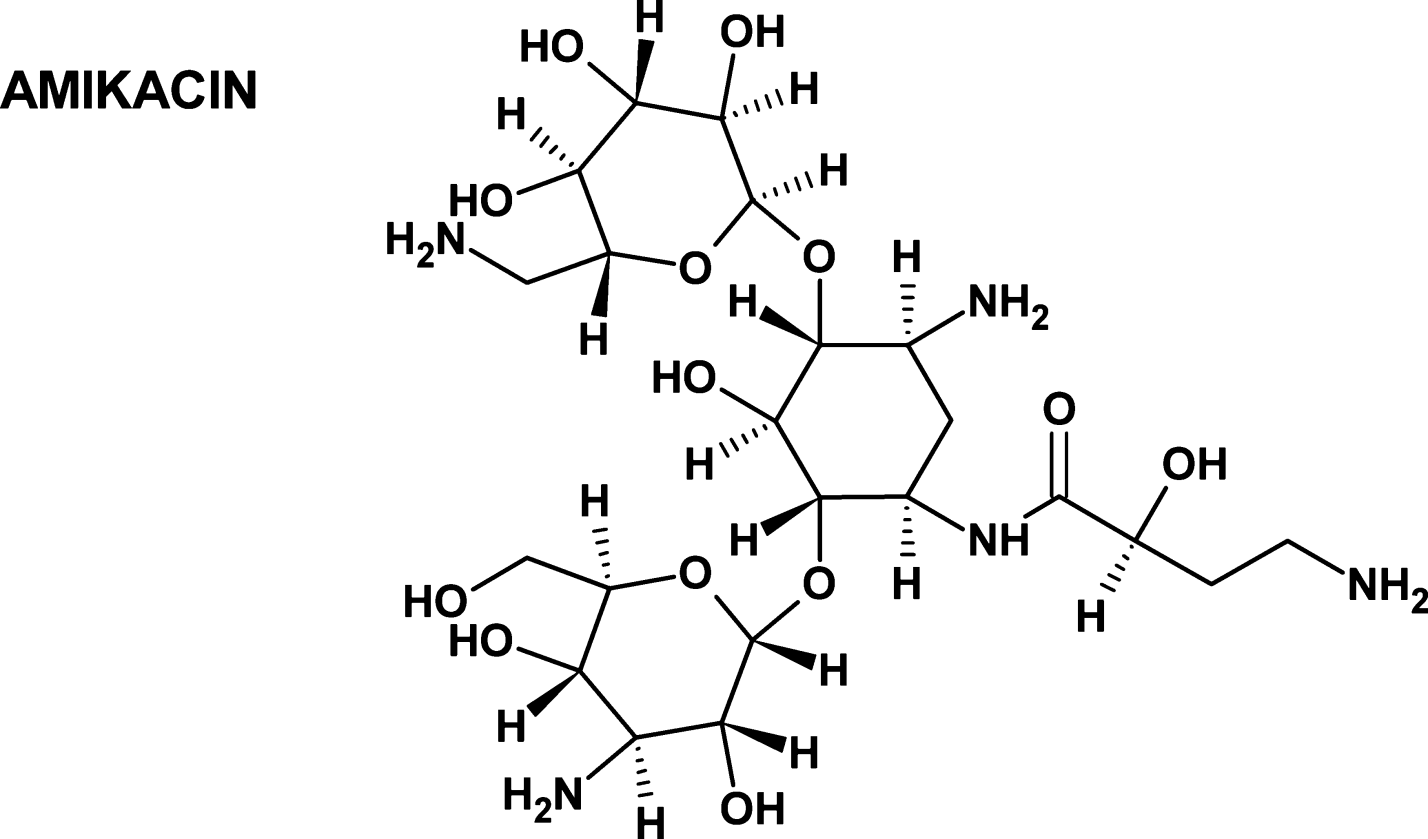
Structure of AMI.

AMI consists of d-glucosamine and d-kanosamine connected with aminocyclitol by glycosidic bonds (Figure 1). It exhibits high polarity and hydrophilicity (the logarithm of the partition coefficient is equal to −7.40), as well as high solubility in water (185 g/L, 25 °C), slight solubility in methanol, and marked insolubility in other organic solvents. Its mean values of constants for acid and basic dissociation are 12.1 and 9.79, respectively.

Polymer-based systems for targeted drug delivery have been widely researched in recent years. They permit dosage of a high concentration of the active substance, localized in the designated tissue at a specific time, without exceeding levels of systemic toxicity. Additionally, it is possible to tailor a polymer matrix to exhibit specific kinetics of release, thereby personalizing therapy for patients. The benefit of this is reduction in the frequency of dosage and mitigation of side effects. Such advantages are especially important and desirable for treatment with AMI.

Microparticles (MP, herein singular and plural) are polymer carriers suitable for use as drug delivery systems. Fabricating MP facilitates encapsulation of the drug molecules within a so-called polymer cage. This not only means the molecules of the active substance are protected physically but the potential also exists for customizing the kinetics of drug release, depending on physicochemical properties of the given materials. The most common procedure for encapsulation involves evaporating solvents in an aqueous emulsion (*e.g.*, water/oil or water/oil/water emulsions). Therefore, the solutions of the drug and polymer are solidified through creating MP (see Figure S1). The fabrication methods are relatively simple and easily performed on an industrial scale.

Either natural or synthetic polymers are applicable for encapsulating a medicinal drug. Examples of the former include proteins, polysaccharides, and peptides, for example, alginate/chitosan particles suitable for hydrophobic drugs such as quercetin^4^ and proteases.^5^ As for synthetic polymers, those commonly employed comprise poly(lactic acid) (PLA), a copolymer of poly(lactic acid) and poly(ethylene glycol) (PLA–PEG), and poly(lactic-*co*-glycolic acid) (PLGA), the latter of which can encapsulate proteins.^6^

Out of these materials, PLA and PLA–PEG make for highly efficient drug carriers with sustainable and long-term release, bolstered by their properties of biocompatibility, biodegradability (by hydrolysis and enzymatic reactions) without risk of hazardous products, general availability, and affordability.^7,8^ Another notable aspect is that PLA is approved by the Food and Drug Administration for medical use.^7^

Some PLA- or PLGA-based systems loaded with aminoglycosides (including AMI) have been reported in the literature,^9−16^ and the ones most widely employed aid in the treatment of respiratory infections and osteomyelitis.^9,10,12−16^ It is worth noting, however, that those loaded with aminoglycosides are difficult to fabricate because of the hydrophobic character of the polymers employed, for example, PLA. The reason is that during emulsification, greater affinity for the drug exists in the external aqueous phase than that for the polymer (Figure S1). Encapsulation heightens the stability of the material structure during treatment and allows for controlled, extended, and gradual release of the active substance, due to the occurrence of degradation and/or swelling in a hydrolytic environment.

As far as the authors are aware, only a few studies have been published on AMI encapsulation within a synthetic polymer. The first study utilized Eudragit RS100 and RL100 polymers and was dedicated to the delivery of an ocular drug.^17^ Another investigated alginate nanoparticles with modified PLGA had been loaded with both AMI and moxifloxacin for the treatment of tuberculosis.^18^ A study by Sabaeifard et al. presented a procedure for encapsulating AMI in PLGA nanoparticles as a drug carrier for treating infections caused by *P. aeruginosa*, reporting a loading efficiency (LE) of 26.0 ± 1.3 μg/mg.^9,16^ In comparison with these,^9,16,17^ the proposed means of encapsulating AMI detailed herein utilizes low-molecular-weight PLA and PLA–PEG supplemented with poly(vinyl alcohol) (PVA) by W/O/W emulsification. The authors concentrated on developing materials with a broad spectrum of antibacterial activity and controlled kinetics of drug release. The novel materials are suitable for medicating against nosocomial infections (including postoperative wounds) and numerous bacterial infections (such as sepsis or tuberculosis). The form of the material, that is, MPs, facilitates the application directly on the infected tissue, for example, *via* a dressing with an appropriately selected dose of AMI-loaded MP, or through administration of an dosed aerosol. The unquestionable advantage consists in the versatile forms of administration, especially in hospital treatment, where infections of various tissues occur.

This study details the comprehensive research conducted on the properties of the fabricated materials, including their antimicrobial action and kinetics of *in vitro* drug release, as well as characterization of their morphology and elemental analysis.

## 2 Experimental Section

### 2.1 Materials

The following were used: amikacin (AMI) disulfate (Interquim); lactic acid (LA) 80% water solution (Merck); tin(II) 2-ethylhexanoate [Sn(Oct)_2_] (Sigma-Aldrich); deionized water; poly(ethylene oxide) (PEG) (*M*_w_ = 380÷420 g/mol) (Merck); PVA 80% hydrolyzed (*M*_w_ = 9000 ÷ 10,000 g/mol) (Merck); chloroform (Chromspec); acetone; methanol (MeOH); acetonitrile (ACN); tetrahydrofuran-stabilized BHT (THF) (Merck); ammonium acetate (Sigma-Aldrich); *o*-phthaldialdehyde (OPA); acetic acid; boric acid (Sigma-Aldrich); sodium hydroxide; potassium hydroxide; sodium chloride; potassium chloride; dipotassium phosphate (Lach-Ner); and monosodium dihydrogen orthophosphate (PENTA). Polystyrene standards [for gel permeation chromatography (GPC)] equaled 580 ÷ 6,000,000 g/mol (Polymer Laboratories Ltd.)

The following bacterial strains (Tomas Bata University in Zlín, Czech Republic) were used: *S. aureus* CCM 4516; *E. coli* CCM 4517; *Enterococcus faecalis* CCM 3956; *K. pneumoniae* CCM 4415; and *P. aeruginosa* CM 1961.

### 2.2 Synthesis of PLA and PLA–PEG

The PLA was synthesized, according to a procedure derived from a method described by Pavelkova et al.^19^ In brief, 100 mL of the LA monomer was poured into a round-bottom two-neck distilling flask that was then connected to a condenser. The mixture was formulated by a dehydration phase stirred in an oil bath at 160 °C under reduced pressure, at 20 kPa for 4 h, and 0.5% w/w of Sn(Oct)_2_ was added in afterward. The pressure was subsequently reduced to 3 kPa, and the reaction continued for 24 h. The product of the polycondensation reaction was precipitated with MeOH and water, purified with acetone, and dried in a vacuum oven.

The synthesis of the PLA–PEG was analogous to that described for the PLA, except 10% w/w PEG and 0.5% w/w of Sn(Oct)_2_ were added in after dehydration. All subsequent steps were identical.

### 2.3 Preparation of the PLA/PVA-AMI and PLA–PEG/PVA-AMI MPs

2% w/v of the synthesized PLA or PLA–PEG was dissolved in chloroform overnight. 15 mL of the resultant polymeric solution was supplemented with 2.5 mL of 2.5% w/v AMI water solution, added dropwise under sonication in an ice bath. The primary emulsion was sonicated for 25 min, following which 100 mL of 1% w/v PVA solution was gradually added into it over the course of an hour. The solution was mixed overnight to evaporate the chloroform and stabilize the particles. The suspension obtained was centrifuged (15 min, 9000 rpm) and washed with deionized water three times, frozen overnight, and lyophilized (Figure S2).

### 2.4 Characterization

#### 2.4.1 Differential Scanning Calorimetry

Characterization of the synthesized PLA and PLA–PEG was carried out on a DSC1 differential scanning calorimeter (Mettler Toledo). The temperature cycle was set as follows: heating from −35 to 200 °C (rate: 10 °C/min), then 2 min heating at 200 °C, and cooling to −35 °C (rate: 10 °C/min); this was repeated after 2 min of maintained temperature. This procedure for the measurement occurred under a nitrogen atmosphere.

#### 2.4.2 Gel Permeation Chromatography

GPC analysis was performed to characterize the synthesized PLA and PLA–PEG and study the degradation of the MP, so as to discern loss in mass in an aqueous environment. GPC transpired as follows: three connected LC columns were used—PL gel MIXED-A (300 × 7.8 mm, 20 μm) + MIXED-B (300 × 7.8 mm, 10 μm) + MIXED-D (300 × 7.8 mm, 5 μm); THF was applied as a mobile phase at the flow rate of 1 mL/min; separation was carried out at the established temperature of 40 °C; and the injection volume equaled 100 μL. For detection purposes, a refractive index detector and viscometric detector were employed. The calibration curves were plotted, according to polystyrene standards (580 ÷ 6,000,000 g/mol, Polymer Laboratories Ltd., UK).

#### 2.4.3 Scanning Electron Microscopy

Scanning electron microscopy (SEM) micrographs of the unloaded and loaded polymer systems (MP of PLA/PVA and MP of PLA–PEG/PVA before and after AMI encapsulation) were obtained on a Nova NanoSEM 450 scanning electron microscope set to 5 kV. The samples were placed on the carbon tape and coated with a sputtered gold/palladium layer (SC7620 Mini Sputter Coater, Quorum Technologies).

#### 2.4.4 Fourier Transform Infrared Spectroscopy–Attenuated Total Reflectance

Fourier transform infrared spectroscopy–attenuated total reflectance (FTIR–ATR) analysis was conducted to gauge whether interaction occurred between the AMI and polymer carriers, on a Nicolet iS5 FTIR spectrometer equipped with an iD5 ATR accessory, at the resolution of 4 cm^–1^, across 64 scans and with Ge as the optical material. The series of analyzed samples comprised AMI sulfate standard (powder), MP of PLA/PVA with and without AMI, and MP of PLA–PEG/PVA with and without AMI.

#### 2.4.5 Elemental Analysis

Elemental studies of the prepared materials as powder were performed *via* energy-dispersive X-ray fluorescence (EDXRF), on a Thermo Scientific ARL Quant X spectrometer. Measurements were taken for CHO, Al, Ca, S, Cl, Mg, K, and Sn; the samples were analyzed in the He measurement mode.

The FLASH analytical method was employed for determining the percentage of C, H, N, O, and S. The destructive Dumass combustion method was applied (960 °C), and the products (CO_2_, N_2_, H_2_O, and SO_2_) of the catalytic reaction were analyzed by gas chromatography with thermal conductivity detection (GC-TCD); the resulting values are given in percent by weight (% w/w). Sulfanilamide was used as a standard. Each sample was prepared in triplicate, and two measurements were conducted to determine the value for SD.

#### 2.4.6 Brunauer–Emmett–Teller Surface Area Analysis

Analysis of the surface and porosity of the materials took place on a Micrometrics Brunauer–Emmett–Teller (BET)-Methodsurface device (Belsorp-mini II, BEL Japan, Inc.). Prior to taking measurements for the MP of PLA/PVA-AMI and PLA–PEG/PVA-AMI, the samples were degassed for 3 h at 40 °C.

#### 2.4.7 Thermogravimetric Analysis

The thermal stability of the materials (MP—unloaded and loaded) was established through thermogravimetric experiments conducted on a thermogravimetric analysis (TGA) Q500 unit (TA Instruments, New Castle, USA) under a nitrogen atmosphere. The heating rate was 10 °C per 1 min, while the range in temperature encompassed 25–500 °C.

#### 2.4.8 Microbiological Properties—Disk Diffusion Method (Kirby–Bauer Method)

Equal masses of the samples (AMI sulfate and MP with and without AMI) were placed on inoculated Mueller-Hinton agar (two samples per Petri dish) and incubated at 35 °C for 18–24 h. After incubation, the width of the inhibition zone for each sample was measured to the nearest millimeter on a SCAN 500 inhibition zone reader (version 8.2.0.0). The samples were analyzed in triplicate.

Testing was performed on the bacterial strains of *S. aureus* CCM 4516, *E. coli* CCM 4517, *E. faecalis* CCM 3956, *K. pneumoniae* CCM 4415, and *P. aeruginosa* CM 1961; the concentration of the bacterial suspensions equaled 10^6^–10^7^ cfu/mL.

#### 2.4.9 High-Performance Liquid Chromatography

High-performance liquid chromatography (HPLC) was carried out for determination of AMI, utilizing “in-needle” derivatization with OPA reagent and fluorescence detection (HPLC Dionex UltiMate 3000 Series, Thermo Fisher Scientific).

The stock solution of AMI (1 mg/mL) was prepared in deionized water and stored at −20 °C. Working standard solutions (0.5, 1, 5, 10, 25, 50, and 100 μg/mL) were prepared daily by diluting the stock solution with phosphate-buffered saline (PBS, pH = 7.4). The PBS was prepared by dissolving 8 g of NaCl, 0.2 g of KCl, 1.44 g of Na_2_HPO_4_, and 0.24 g of KH_2_PO_4_ in 1 L of distilled water, and the pH of the buffer was adjusted to 7.4 by NaOH.

Chromatographic separation was carried out using Waters XSELECT CSH C18 (4.6 × 250 mm, 5 μm) column equipped with a security guard column (Phenomenex) at 30 °C. A mobile phase mixture of 100 mM acetate buffer (pH 5.8, eluent A) and ACN (eluent B) was used under isocratic conditions (55:45 v/v) at a flow rate of 0.4 mL/min; the duration of analysis was 20 min, and the sampler was set to 7 °C. The volume of the injection was defined by user-defined program settings; 10 μL was injected into the column which originated from a drawn sample volume of 1 μL.

The reagents introduced into the needle during derivatization comprised 1 μL of sample, 5 μL of borate buffer (I), 3 μL of OPA reagent (III), and 1 μL of 1 M acetic acid, where

I Buffer solution: 5 g of H_3_BO_3_ + 90 mL of H_2_O; made up to the volume of 100 mL with KOH 47% to pH 11
II Reducing solution: 250 μL of 2-mercaptoethanol + 10 mL of buffer solution (I)
III OPA reagent: 2.5 mg of OPA + 400 μL of MeOH + 200 μL of reducing solution (II) + 4.4 mL of buffer solution (I)

Detection was conducted with 330 and 440 nm as the excitation and emission wavelengths, respectively. An external calibration method was applied to quantify the AMI. The calibration curve obtained was linear (R^2^ = 0.9994) within the given ranges of the concentration. The limits of detection (LOD = 3.3 × calibration curve intercept standard deviation divided by the slope of the calibration curve) and the limits of quantitation (LOQ = 3 × LOD) were established as follows: LOD = 0.19 μg/mL and LOQ = 0.58 μg/mL.

#### 2.4.10 In Vitro Release Study into Liquid Media

For the release test, 50 mg of the MP were immersed in 10 mL of PBS (pH 7.4). The samples were incubated at 37 °C with gentle shaking (100 rpm). 1 mL of the liquid fraction of each sample was collected (at a specific time) and replaced with a portion of fresh buffer. Each sample was prepared in triplicate and analyzed three times by HPLC. The test was carried out under sink conditions, whereby AMI was characterized by complete solubility under the applied conditions.

Liquid fractions following preparation of the MP were also investigated to gauge their encapsulation efficiency (% EE, eq 1) and % LE (eq 2)
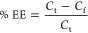
1
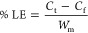
2where *C*_t_—the total amount of AMI used to modify the MP (mg); *C*_f_—the amount of excess AMI in the waste solution after the material had been prepared (mg); and *W*_m_—the weight of the dry mass of the prepared material (mg).

The cumulative release (CR) and cumulative release in percent (% CR) of AMI were calculated as follows (eqs 3 and 4)
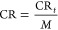
3where *CR*_*t*_—the amount of AMI (mg) released at time *t* and *M*—the mass of the polymer sample extracted for analysis (g).
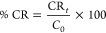
4where *C*_0_—the amount of AMI loaded (mg).

The kinetics of AMI release were also researched. In this context, the release constant *K* was established through different mathematical models—zero-order kinetics (eq 5), first-order kinetics (eq 6), the HiguChi square root model (eq 7), and the Korsmeyer–Peppas model (eq 8)
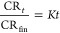
5
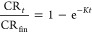
6
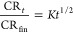
7
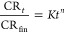
8where CR_fin_—the total amount of AMI released (mg); *K*—the release constant; *n*—the diffusion coefficient (where *n* ≤ 0.45 represents Fickian diffusion, and 0.45 < *n* < 1 is the characteristic for anomalous transport^20^).

## 3 Results and Discussion

### 3.1 Characterization of the Synthesized PLA and PLA–PEG

Determining the primary properties of the prepared PLA and PLA–PEG after synthesis (polycondensation) was performed by differential scanning calorimetry (DSC; the results are given in Table 1) and GPC.

**Table 1:** DSC Results for the Synthesized PLA and PLA–PEG^a^

sample	*T*_g_ (°C)	*T*_m_ (°C)	Δ*H*_m_ (J/g)	*T*_c_ (°C)	Δ*H*_c_ (J/g)
PLA	41.1	143.4	43.5	94.5	19.9
PLA–PEG	32.5	139.3	44.7	84.7	34.4

a *T*_g_—temperature of glass transition; *T*_m_—melting point; Δ*H*_m_—enthalpy of fusion; *T*_c_—temperature of crystallization; and Δ*H*_c_—enthalpy of crystallization.

The PLA–PEG copolymer exhibited reduction in thermal stability compared to PLA, in addition to which crystallization for the former occurred at a temperature of less than 10 °C, unlike the latter, and its content of the crystalline phase was higher by approximately 15 J/g.

Upon further analysis, it was noted that the PLA–PEG copolymer demonstrated a lower glass-transition temperature (*T*_g_) than PLA, potentially caused by the low *T*_g_ value for pure PEG in comparison to PLA.^21,22^ Also of interest was a decrease in *T*_g_ attributed to a drop in the molecular weight and rise in the number of functional groups at the end of the polymer chain; hence, a reduced value of *T*_g_ for the synthesized PLA–PEG copolymer was recorded (see Table 1).^19^ Adding PEG raised the values of Δ*H*_m_ and Δ*H*_c_, a finding reported in the literature as possibly pertaining to increase in the crystallinity of the resultant copolymer.^21,23,24^ Such heightened crystallinity due to the presence of PEG may slow down the release of the drug from the polymer matrix, although PEG also has the effect of raising the amphiphilicity of the material and, subsequently, the affinity of AMI for the polymer matrix (*i.e.*, an increase in LE). An additional consequence of supplementing with PEG is that the reduction in *T*_g_ could positively affect the degradation of the polymer at the stage of synthesis of the MP and their use afterward (see the degradation study in the following section and Table 10).

As for other considerations related to the crystallization process, it should be noted that the literature states that aminoglycosides, such as AMI, do not significantly affect the crystallization of PLA- and PEG-based materials.^24^ Therefore, only the PLA and PLA–PEG polymers (after synthesis) underwent DSC.

A review of the literature revealed that the masses of PLA material prepared by polycondensation varied, according to the given reaction conditions, especially the type of catalyst employed. In this context, the values of number average molar masses (*M*_n_) for PLA reported therein ranged from 2000–8000 g/mol, these being directly affected by the catalyst used.^25^ The intention of the authors of this study was to obtain materials of a relatively low-molecular weight (3000–4000 g/mol), and a modified methodology described by Pavelkova et al. was investigated for this express purpose.^19^ They prepared a PLA copolymer with 7.5% w/w PEG and a number average molar mass (*M*_n_) of 3200 g/mol. The material, after modification, was capable of controlled delivery of a bioactive agent (metazachlor). Table 2 summarizes the GPC results obtained herein for the given synthesized materials, showing that PLA and the PLA copolymer with 10% w/w PEG possessed number average molar masses (*M*_n_) of 3000 and 2800 g/mol, respectively.

**Table 2:** GPC Results for the Synthesized PLA and PLA–PEG^a^

sample	*M*_w_ (g/mol)	*M*_n_ (g/mol)	*M*_p_ (g/mol)	*D̵* (−)
PLA	5200	3000	4600	1.73
PLA–PEG	3900	2800	3500	1.39

a *M*_w_—weight average molar mass; *M*_n_—number average molar mass, *M*_p_—molecular weight of the highest peak; and *D̵*—polydispersity index (*D̵* = *M*_w_/*M*_n_).

The materials (PLA and PLA–PEG) were both applied to obtain MP loaded with AMI.

### 3.2 MPs Loaded with AMI: PLA/PVA-AMI and PLA–PEG/PVA-AMI

The MP loaded with AMI were prepared *via* the water/oil/water (W_1_/O/W_2_) emulsion method, utilizing AMI (W_1_) and PVA (W_2_) aqueous solutions and PVA as the surfactant. It has been reported in the literature that PLA supplemented with PVA enhances the properties of PLA-based materials through increase in hydrophilicity and ductility.^26^ The organic component (O) consisted of PLA or PLA–PEG in chloroform.

**Figure 2:**
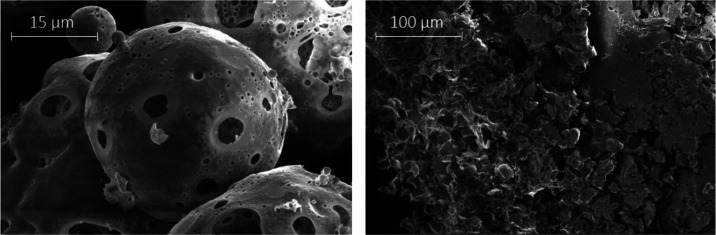
SEM images of fabricated MP based on the given methodology:^27^ PLA/PVA-AMI (left) and PLA–PEG/PVA-AMI (right).

A preliminary study investigated a methodology described by Rafat et al. on MP based on PLA–PEG and loaded with protein^27^ to determine the potential it had for fabricating materials with AMI (see Supporting Information: *description of initial conditions for preparation of the MPs*). SEM was conducted to characterize the prepared MP at the outset, the images revealing the MP prepared with PLA possessed a spherical, “moon-like” shape of *ca.* 45 ± 10 μm in diameter. In contrast, amorphous aggregates were formed when PLA–PEG was used instead (see Figure 2), which resembled those obtained and described as MPs by Rafat et al.^27^

**Figure 3:**
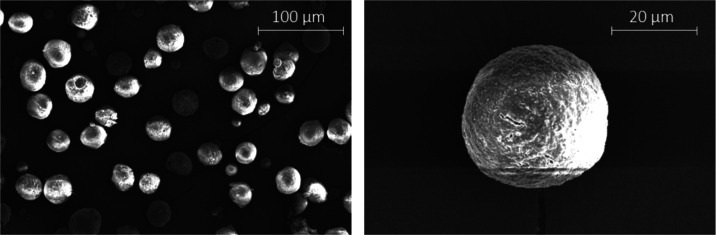
SEM images of the PLA/PVA-AMI particles.

**Figure 4:**
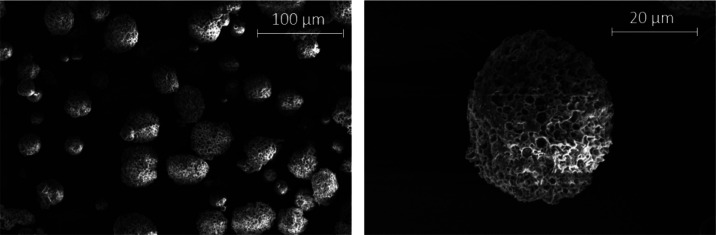
SEM images of the PLA–PEG/PVA-AMI particles.

An attempt was made to improve the spherical shapes of the MP (especially for PLA–PEG) through extending the time of dissolution of PLA and PLA–PEG in chloroform. It was observed that after 24 h of mixing, as opposed to 3 h as previously described, the MP of PLA–PEG took on a more spherical appearance, yet large aggregates were still present. Other modifications included altering the proportions of the solutions applied (the water and organic solutions during the fabrication of the emulsions), further homogenization on a mechanical mixer when preparing the emulsions, and a stabilization phase (see Section 2.3). These brought about significant improvement in MP formation (Figures 3 and 4); the samples exhibiting diameters of 27.4 ± 6.7 μm for MP PLA/PVA-AMI and 31.9 ± 7.0 μm for MP PLA–PEG/PVA-AMI. In terms of the medical application, MP with diameters of up to 40 μm are suitable for use in suspensions intended for the injection or in the form of an aerosol for respiratory ailments. In light of this, research efforts continued as a result of the MP fabricated through the optimized methodology.

**Figure 5:**
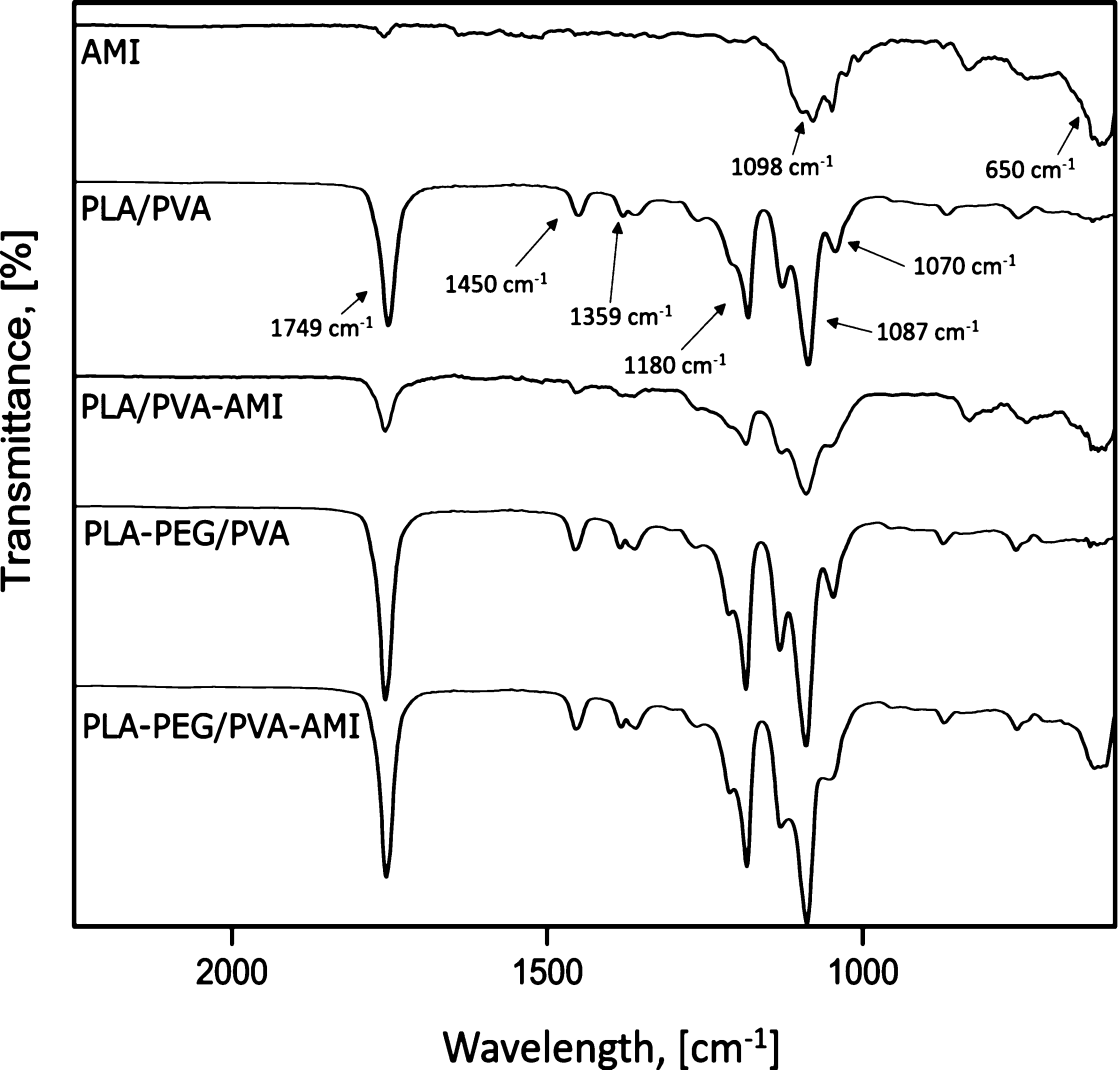
FTIR spectra for the fabricated MP before and after being loaded with AMI.

FTIR spectroscopy was performed to confirm the successful loading of MP PLA/PVA and MP PLA–PEG/PVA with AMI. Measurements were taken in the range of 4000–600 cm^–1^, and Figure 5 provides the details of wavelengths from 2250 to 600 cm^–1^ in order to clearly show the individual FTIR spectra.

Regarding the PLA–PEG/PVA sample without loaded MP, −CH bending vibration peaks are visible at 1359 and 1310 cm^–1^, while that for −CH_2_ appears at 1450 cm^–1^. Comparing the results with the neat PEG spectra^28^ revealed that these peaks indicated the presence of PEG grafts in PLA; note that the occurrence of PLA methyl groups and PEG methylene groups implies that PLA–PEG copolymerization could have taken place. The existence of ester groups is confirmed by a peak at 1749 cm^–1^ (C=O stretching vibrations).

PLA-/PVA-based materials have a characteristic peak at 1750 cm^–1^ (C=O stretching vibrations) and ∼1090 cm^–1^ (a C–O stretch contributing to alcohols).^29^ The other absorbance peaks pertained to −CH bending regions at ∼1450 cm^–1^ (−CH_3_ asymmetric) and ∼1360 cm^–1^ (−CH_3_ symmetric).^30^ MP PLA/PVA and MP PLA–PEG/PVA demonstrated absorption peaks in the same regions (wavelengths).

The characteristic absorption band for AMI sulfate (as a powder standard) is observed in the range 1250–900 cm^–1^.^31^ The peaks reaching a maximum in this region at approximately 1100 cm^–1^ relate to the presence of a sulfate counter-ion (SO_4_^2–^ asymmetric stretching vibrations). The absorption band exhibited between 1200 and 1000 cm^–1^ represents the C–O stretching bands of carbohydrates.^32^ Lastly, the peak at 650 cm^–1^ corresponds to N–H single bond stretching vibrations. Comparing the spectra for AMI and MP prior to and following encapsulation revealed that characteristic bands for AMI were masked by the components of the polymers, meaning that visible changes were only seen at 650 cm^–1^.

Elemental analyses of the loaded and unloaded MP were performed to determine the composition of the drug delivery systems. In the case of EDXRF (Table 3), it was found that all the samples contained an approximate amount of C, H, O, and N elements (as a sum). The reference of pure AMI used for modification purposes contained approx. 12% w/w of S, due to the AMI sulfate salt antibiotic loaded. The remaining materials under investigation did not contain S, as a consequence of AMI bonding with the polymer matrix and the materials being washed after fabrication, whereby sulfates were removed. Every type of MP contained Al at a similar level (approx. 2% w/w), potentially caused by the apparatus employed to prepare them; a characteristic of the pure AMI standard sample was a lack of Al. The portion of elements such as Ca, S, K, and Cl in all the analyzed samples was negligible, below the LOQ (<0.5% w/w). In addition, no Sn was detected, as present in the catalyst during polycondensation. Three samples (MP PLA/PVA, MP PLA–PEG/PVA, and PLA–PEG/PVA-AMI) exhibited Mg at approximately the level of quantification (0.5% w/w), and one sample (AMI) showed 1% w/w.

**Table 3:** Results of Elemental Analyses (EDXRF) of the Materials in the Form of MP

sample	CHON (%)^a^	Al (%)	S (%)	Mg (%)
MP PLA/PVA	96.80	2.38		0.52
MP PLA/PVA-AMI	97.90	2.00		
MP PLA–PEG/PVA	96.50	2.34		0.58
MP PLA–PEG/PVA-AMI	96.30	2.16		0.54
AMI	86.40		12.60	1.00

a weight in percent.

In order to gauge the content of single elements (N, C, H, and S), the Arc Flash method was applied (Table 4). In consideration of the S present, the results obtained were largely in agreement with EDXRF analysis. It was observed that the materials loaded with AMI were characterized by a higher content of nitrogen. As for the MP loaded with AMI, nitrogen content increased by *ca.* 1.2% w/w for both types of MP (PLA/PVA-AMI and PLA–PEG/PVA-AMI) in comparison to the unloaded MP. These results suggest the presence of AMI after drug loading had taken place, as indicated by the amino groups in the AMI molecule. The amounts of C and H were similar for the loaded and unloaded MP, at 49% w/w (C) and 5% w/w (H).

**Table 4:** Results of Elemental Analyses (Arc Flash) of the Materials in the Form of MP

sample	N ± SD^a^ (%)^b^	C ± SD (%)	H ± SD (%)	S ± SD (%)
MP PLA/PVA	1.84 ± 0.41	49.17 ± 0.28	5.81 ± 0.23	
MP PLA/PVA-AMI	3.06 ± 0.55	47.68 ± 0.74	5.52 ± 0.20	
MP PLA–PEG/PVA	2.92 ± 0.66	49.26 ± 0.35	5.93 ± 0.29	
MP PLA–PEG/PVA-AMI	4.14 ± 0.26	49.319 ± 0.044	5.17 ± 0.38	
AMI	8.114 ± 0.010	30.18 ± 0.22	6.364 ± 0.072	11.5 ± 1.2

a SD—three independently prepared material samples, analyzed twice.

b weight in percent.

According to the results of BET analysis (Table 5), the MP PLA–PEG/PVA-AMI were characterized by a slightly greater surface area (1.929 m^2^/g) than PLA/PVA-AMI (1.161 m^2^/g), arising through the more irregular structure of MP PLA–PEG/PVA-AMI (see Figure 4). Additionally, the mean pore diameter of the PLA–PEG-based MP was approximately 14 nm less than that for PLA-based ones.

**Table 5:** Results of BET Analysis for the MP

sample	surface area (m^2^/g)	mean pore diameter (nm)	total pore volume (*p*/*p*_0_ = 0.990) (cm^3^/g)
MP PLA/PVA-AMI	1.161	27.739	0.008
MP PLA–PEG/PVA-AMI	1.929	14.081	0.007

**Figure 6:**
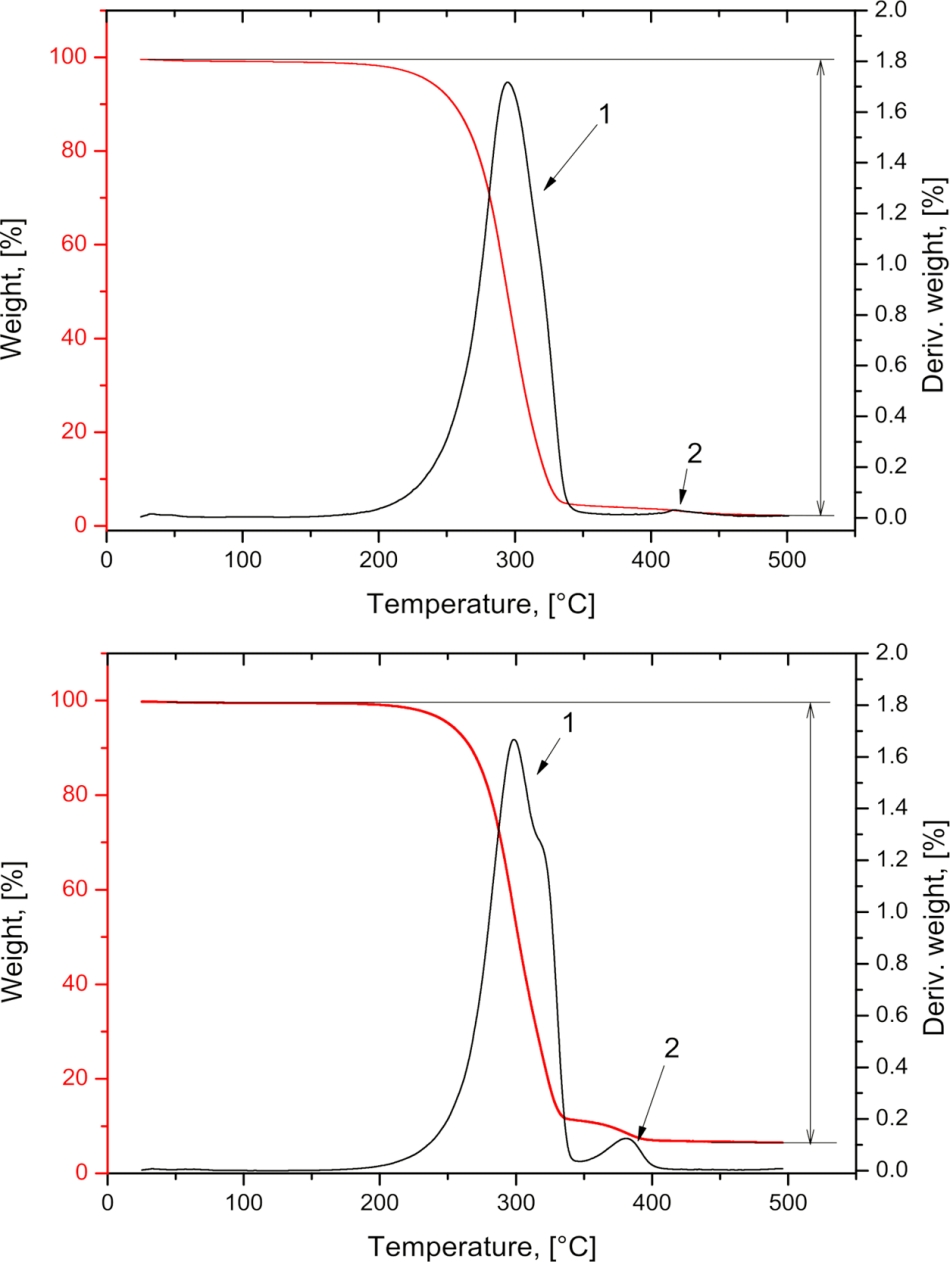
Thermogravimetry and differential thermogravimetry curves for MP PLA/PVA-AMI (upper) and MP PLA–PEG/PVA-AMI (lower).

The thermal stability of the MP was investigated by analyzing TGA. The findings (Figure 6, Table 6) revealed that the fabricated MP had the highest rates for mass loss at 294 and 299 °C for PLA/PVA-AMI and PLA–PEG/PVA-AMI, respectively. In the case of MP PLA/PVA-AMI, the temperature of degradation (183 °C) occurred 17 °C lower than that for MP PLA–PEG/PVA-AMI (Figure 6, peak 1). It is worth noting that the melting points of the materials employed in fabricating the MP were in the range of 160–200 °C (PLA_5000g/mol_ = 160 °C, PVA_10,000g/mol_ = 200 °C, and AMI = 203 °C). According to the literature, a slight loss in mass at 50–150 °C was caused by the evaporation of highly volatile components.^33^ Loss in mass at *ca.* 300–400 °C indicated the decomposition of PLA.^34^ In contrast, the PVA decomposed at 400–450 °C.^33^ The thermal decomposition of PEG began at approximately 240 °C,^35^ and AMI probably decomposed at 200 °C.

**Table 6:** Results of TGA Analysis for the MP; the Peak Numbers According to Figure 6^a^

	peak 1				peak 2							
sample	*T*_i_ (°C)	*T*_m_ (°C)	*T*_f_ (°C)	Δ*w* (%)	*T*_i_ (°C)	*T*_m_ (°C)	*T*_f_ (°C)	Δ*w* (%)	Δ*w*_fin_ (%)	Δ*T*_5%_ (°C)	Δ*T*_10%_ (°C)	Δ*T*_50%_ (°C)
MP PLA/PVA-AMI	183	294	356	94.6	356	422	498	2.0	96.6	236	255	294
MP PLA–PEG/PVA-AMI	200	299	343	87.5	343	381	400	4.3	91.8	251	267	302

a *T*_i_—onset temperature; *T*_m_—temperature corresponding to the maximum rate of mass loss; *T*_f_—final temperature; Δ*w*—mass loss in the range *T*_i_–*T*_f_; Δ*w*_fin_—total mass loss; and Δ*T*_5%_, Δ*T*_10%_, and Δ*T*_50%_—temperature corresponding to 5, 10, and 50% of sample mass loss, respectively.

The temperatures corresponding to 5, 10, and 50% w/w of mass loss for the samples were established (Δ*T*_5%_, Δ*T*_10%_, and Δ*T*_50%_, respectively). It was found that MP PLA–PEG/PVA-AMI exhibited the greatest thermal stability; its temperatures of degradation for specified points of mass loss were higher by approx. 8–15 °C in comparison with MP PLA/PVA-AMI. These results proved that the fabricated MP possessed thermal stability, making it possible to further reprocess them with other techniques, for example, with the aim of obtaining a different kind of material.

In relation to antimicrobial properties (Table 7), the findings showed that both types of MPs (PLA/PVA-AMI and PLA–PEG/PVA-AMI) exerted an antimicrobial effect against most of the investigated strains (except *E. faecalis*). Interestingly, MP PLA/PVA-AMI demonstrated better activity against *S. aureus* and *P. aeruginosa* strains (approximately twice that of PLA–PEG/PVA-AMI). This suggests that adding the copolymer PLA–PEG diminished the antimicrobial properties through possible interactions with the functional groups of AMI, causing changes in the mechanism of treatment and inhibition of drug action. The MPs without AMI were characterized by an absence of antimicrobial activity against the bacterial strains.

**Table 7:** Antibacterial Activity of the Fabricated MP

	width of the inhibition zone ± SD^a^ (mm)				
sample	*S. aureus*	*E. coli*	*E. faecalis*	*P. aeruginosa*	*K. pneumoniae*
PLA/PVA-AMI	8.0 ± 0.0	8.5 ± 0.5	0.0	6.5 ± 0.5	10.0 ± 0.0
PLA/PVA	0.0	0.0	0.0	0.0	0.0
PLA–PEG/PVA-AMI	3.5 ± 0.5	5.5 ± 0.5	0.0	3.0 ± 0.0	7.0 ± 0.0
PLA–PEG/PVA	0.0	0.0	0.0	0.0	0.0
AMI	16.5 ± 0.5	16.5 ± 0.5	11.0 ± 0.0	19.0 ± 0.0	18.0 ± 0.0

a SD—three independently prepared material samples, analyzed three times.

**Figure 7:**
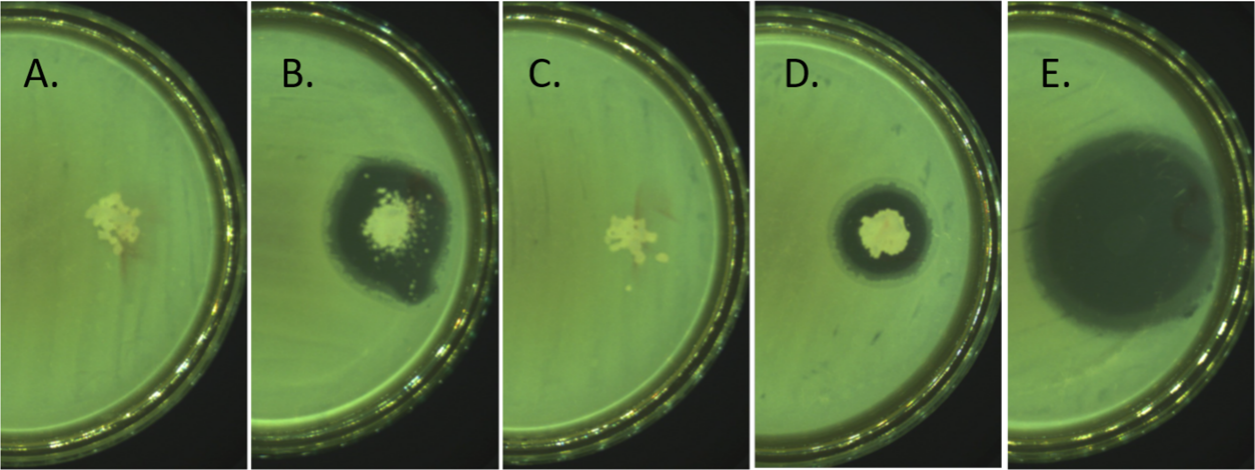
Examples of inhibition zones (against *E. coli*): (A)—pure PLA/PVA; (B)—PLA/PVA-AMI; (C)—pure PLA–PEG/PVA; (D)—PLA–PEG/PVA-AMI; and (E)—pure AMI.

Further investigation included estimation of the % EE (eq 2) and % LE (eq 3) of the MP. The results presented in Table 8 show that PLA–PEG/PVA-AMI demonstrated approximately twice as high % EE (32%) and three times as high % LE (10.7%). This could have been due to enhancement of the amphiphilic nature of the material through adding PEG, potentially leading to a heightened affinity of AMI for the polymer matrix. However, the diameters of inhibition zones (Figure 7) were smaller in comparison with PLA/PVA-AMI, which lends support to the study previously described on reduction in antimicrobial properties through applying the copolymer PLA–PEG instead of PLA.

**Table 8:** EE and LE of the MP Loaded with AMI (Based on Eq. 1 and 2)

sample	EE ± SD^a^ (%)	LE ± SD (%)
PLA/PVA-AMI	16.3 ± 1.3	3.65 ± 0.15
PLA–PEG/PVA-AMI	32.0 ± 4.8	10.7 ± 3.3

a SD—three independently prepared material samples, analyzed three times.

As mentioned earlier, only a few studies on AMI encapsulation in a synthetic polymer (PLGA) were found in the literature. Compared to the findings from the literature on the EE of a PLGA system loaded with AMI (76.77 ± 3.81%),^9,16^ the values summarized in Table 8 of this work are lower. In the methodology presented here, however, a significant excess of the drug was used during preparation of the MP (W_1_ solution) due to the greater affinity of AMI to the aqueous phase; nevertheless, the low cost of AMI means that there is no significantly increase in expenditure on materials. A notable aspect, though, is that the LE, calculated as μg of AMI per 1 mg of the polymer, was determined at the following levels: 36.5 ± 1.5 μg/mg for the PLA-based MP and 106 ± 32 μg/mg for PLA–PEG-based MP. These values were higher than those recorded by Sabaeifard et al. (26.0 ± 1.3 μg/mg nanoparticles).^16^ A study by Prior et al.^36^ proposed PLA/PLGA MP loaded with a different aminoglycoside antibiotic—gentamicin, reporting a maximal LE of 8.3 mg/g for PLA/PLGA and only 3.7 mg/g for PLA. These examples show the difficulties that exist in fabricating effective drug delivery systems with polar active substances. The findings described herein prove that the procedure proposed and described for fabricating MP guarantees a higher LE.

**Figure 8:**
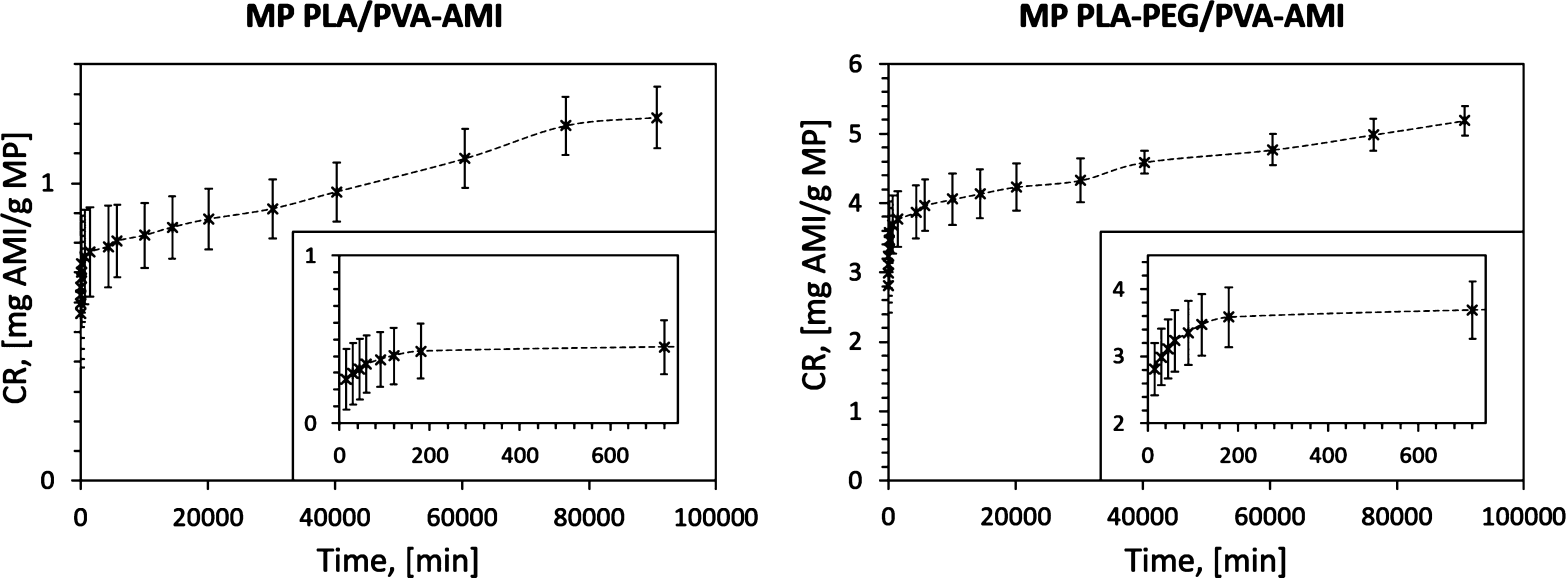
AMI release from MP PLA/PVA and MP PLA–PEG/PVA (SD—three independently prepared material samples).

Considering the *in vitro* release of AMI (Figure 8 and Table S1), the total amounts of antibiotic released from the polymer matrix (mg/g) after 63 days (CR, eq 1) were, respectively, 1.22 ± 0.10 mg/g (% CR = 3.3% w/w, where % CR corresponds to the amount of drug dispensed over a specific time per amount of drug loaded, eq 2) for PLA/PVA-AMI and 5.19 ± 0.21 mg/g (% CR = 4.0% w/w) for PLA–PEG/PVA-AMI. After the first 15 min of testing, the amounts of AMI released equaled 0.56 ± 0.18 mg/g (% CR = 1.5% w/w) for PLA/PVA and 2.81 ± 0.38 mg/g (% CR = 2.2% w/w) for PLA–PEG/PVA.

It is worth noting that the proposed polymer systems provided AMI release even after 2 months, making them suitable for long-term application. In comparison, the established release times of AMI in the literature were 24 h from PLGA^16^ and 12 h from nanoparticles prepared from Eudragit RS 100 and RL 100.^17^

In following investigation, the results obtained were applied to determine the theoretical kinetics of AMI release from the polymer matrix. Table 9 summarizes the established release constant *K*, as calculated by different mathematical models. According to the value for the correlation coefficient (*R*^2^) between findings from analysis of real-word samples and theoretical ones (determined by mathematical assumption), it was clear that the Korsmeyer–Peppas model showed the best match for both forms of fabricated MPs (PLA/PVA-AMI and PLA–PEG/PVA-AMI). Furthermore, based on the value for diffusion convection (*n*, eq 8), it was specified that the mechanism of AMI release was based on Fickian diffusion (*n* ≤ 0.45). This evidences the rapid release of the drug in the first few hours of analysis. This phenomenon was expected due to the stronger affinity of AMI to the aqueous solution (during emulsification). Consequently, the most rapid drug release in the first 3 h was associated with the presence of AMI molecules on or trapped near the surface of the MP. The slower release of AMI in subsequent hours was due to the presence of drug molecules in the deeper layers of the MP, as well as polymer erosion and hydrolytic bulk degradation.^12,16^ Rapid release of a drug followed by a continual, slower trend is preferred for materials with antimicrobial applications.^16^

**Table 9:** Release Constants (*K*) and Correlation Coefficients (*R*^2^) Determined by Mathematical Models

	0-order release		I-order release		HiguChi model		Korsmeyer–Peppas model	
sample	*K*_0_ ± SD^a^	*R*^2^	*K*_I_ ± SD	*R*^2^	*K*_H_ ± SD	*R*^2^	*K*_KP_ ± SD	*R*^2^
MP PLA/PVA-AMI	0.262 ± 0.039	0.9457	0.388 ± 0.082	0.7340	0.245 ± 0.031	0.9740	0.564 ± 0.046	0.9973
MP PLA–PEG/PVA-AMI	0.324 ± 0.014	0.8859	0.539 ± 0.040	0.8158	0.300 ± 0.013	0.9566	0.631 ± 0.028	0.9996

a SD—three independently prepared material samples, analyzed three times.

Despite the similar fit of the HiguChi model (eq 7), it was found that the obtained release constants in the given series of measurements varied greatly from one another (CV > 100%, where CV—coefficient of variation). Consequently, the assumptions of the authors were not met.

A GPC study after 56 days of MP exposure in PBS solution was also carried out. Samples were treated under the same conditions as in the *in vitro* release test. Subsequently, 1 mL of the liquid fraction with suspended MP was collected for each of the prepared samples. To prove the occurrence of MP degradation, reference samples of MP, that is, without exposure to the PBS solution, were also prepared and tested by GPC. Table 10 summarizes the results. Based on the determined parameters, it was found that both types of MPs exhibited reduction in *M*_w_ during the degradation test. In the case of MP PLA/PVA-AMI, the value for *M*_w_ after 56 days of exposure was 2.1 times lower (in comparison to the reference sample, *M*_w_ degradation = 52%). The greatest reduction in *M*_w_ was observed for PLA–PEG/PVA-AMI, where *M*_w_ was 3.6 times lower than that for the sample not exposed to the PBS solution (*M*_w_ degradation = 73%). The observed loss in mass loss indicates a degradation of all the fabricated materials, which is a positive effect and provides for the possibility of release of any trapped drug.

**Table 10:** GPC Results After 0 and 56 days of Degradation in PBS Solution

sample	*M*_w_ (g/mol)	*M*_n_ (g/mol)	*M*_p_ (g/mol)	*D̵* (−)
MP PLA/PVA_0^a^	5800	3800	4800	1.53
MP PLA/PVA-AMI (after 56 days)	2800	1500	750	1.75
MP PLA–PEG/PVA_0	4500	3300	3300	1.36
MP PLA–PEG/PVA-AMI (after 56 days)	1234	975	882	1.79

a Sample_0—reference sample (without immersion in the PBS solution), *M*_w_—weight average molar mass; *M*_n_—number average molar mass, *M*_p_—molecular weight of the highest peak; and *D̵*—polydispersity index (*D̵* = *M*_w_/*M*_n_).

The investigation over the long-term for determining the kinetics of AMI release and degradation of the fabricated materials was necessary due to the expected *burst effect* indicated by the hydrolytic degradation of PLA and relaxation of the polymer chain.

## 4 Conclusions

Encapsulating AMI in synthetic polymers such as PLA or PLA–PEG is a challenge, due to the polarity of AMI, also in comparison to other aminoglycosides like gentamicin or tobramycin. This phenomenon has a significant impact on the drug LE through partial entrapment of the drug in the polymer phase.

Herein, a new emulsification method is proposed with low-molecular-weight PLA or PLA–PEG supplemented with PVA for AMI encapsulation. The resultant MPs were characterized as having diameters of less than 30 μm. The values of LE for the fabricated MP, determined as μg of AMI per 1 mg of the polymer, were as follows: 36.5 ± 1.5 μg/mg for PLA/PVA-AMI and 106 ± 32 μg/mg for PLA–PEG/PVA-AMI. The most rapid AMI release in both cases (MP PLA/PVA and MP PLA–PEG/PVA) was observed within the first 3 h with release kinetics based on the Korsmeyer–Peppas model. The remaining amount of AMI continued to be gradually released even after 63 days. The fabricated materials exhibited high antimicrobial activity against the *S. aureus*, *E. coli*, *P. aeruginosa*, and *K. pneumoniae* bacterial strains. The AMI-loaded MPs did not act against *E. faecalis*, unlike neat AMI. Additionally, due to the high thermal stability of the prepared MP, the potential exists to reprocess them further, for example, by 3D printing tailored, functional drugs in a form desirable for therapy.

## Supporting Information Available

Description of initial conditions for preparation of the MPs; scheme of AMI encapsulation and MP preparation; and AMI release from the PLA/PVA and PLA–PEG/PVA MPs—summarized data (PDF)
