# Supplementary material for: Encapsulation of Amikacin into Microparticles Based on Low-Molecular-Weight Poly(lactic acid) and Poly(lactic acid-co-polyethylene glycol)
Source: Mol Pharm. 2021 Jul 1;18(8):2986–96. doi: 10.1021/acs.molpharmaceut.1c00193 (PMC8397404; doi:10.1021/acs.molpharmaceut.1c00193)
Supplement: Supplementary file 1 — mp1c00193_si_001.pdf [file mp1c00193_si_001.pdf]

SUPPORTING INFORMATION

Encapsulation of Amikacin into Microparticles based on Low-Molecular-Weight Poly(lactic Acid) and Poly(lactic acid-co-polyethylene Glycol)

Marta Glinka[*a], Katerina Filatova[b], Justyna Kucińska-Lipka[c], Eva Domincova Bergerova[b], Andrzej Wasik[a], Vladimir Sedlařík[b]*

[a]Department of Analytical Chemistry, Faculty of Chemistry, Gdańsk University of Technology, G. Narutowicza 11/12, 80-233 Gdańsk, Poland

[b]Centre of Polymer Systems, University Institute, Tomas Bata University in Zlín, Tomáše Bati 5678, 76001 Zlín, Czech Republic

[c]Department of Polymers Technology, Faculty of Chemistry, Gdańsk University of Technology, G. Narutowicza 11/12, 80-233 Gdańsk, Poland

*Corresponding author: marglink@student.pg.edu.pl

Description of initial conditions for preparation of the microparticles

2 mL of 0.5% w/v AMI water solution was dispersed in 6 mL of 2% w/v solution of synthesized PLA or PLA-PEG in chloroform under sonication (10 minutes). This primary emulsion was gradually

added into 40 mL of 1% w/v aqueous PVA solution. The mixture was sonicated per 10 minutes and mixed overnight for evaporation of the organic solvent. Microparticles were collected by centrifugation (15 min, 9000 rpm), washed three times with deionized water, frozen and lyophilized.

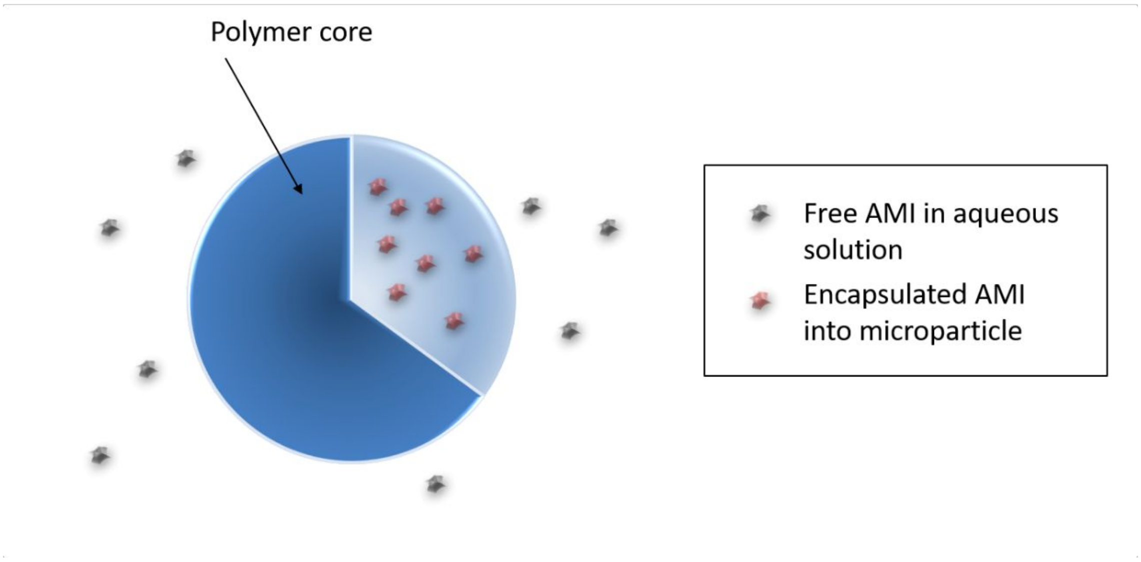

Figure S1. Scheme of amikacin encapsulation.

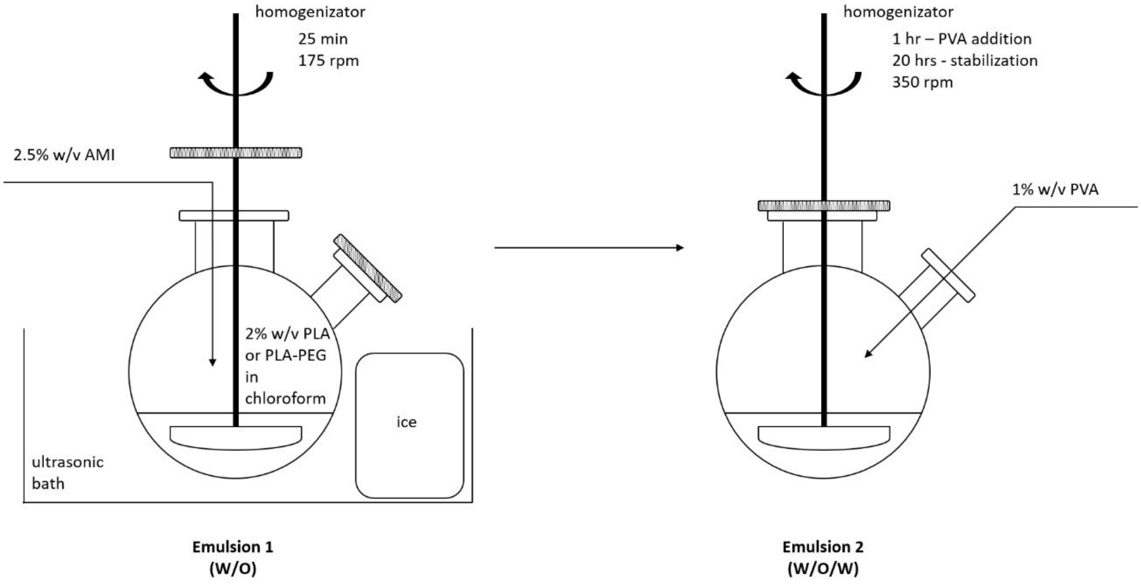

Figure S2. Generalised scheme of microparticles preparation.

Table S1. AMI release form PLA/PVA and PLA-PEG/PVA microparticles (MP). CR and %CR determined using Eq. 1 and 2.

	MP PLA/PVA-AMI			MP PLA-PEG/PVA-AMI		
Time, [min]	%CR ± SD[1], [%]	CR ± SD, [mg AMI/g MP]	CV[2], [%]	%CR ± SD, [%]	CR ± SD, [mg AMI/g MP]	CV, [%]
15	1.50 ± 0.49	0.56 ± 0.18	32.4	2.16 ± 0.30	2.81 ± 0.38	13.7
30	1.59 ± 0.49	0.60 ± 0.18	31.1	2.30 ± 0.33	2.99 ± 0.42	14.2
45	1.66 ± 0.48	0.62 ± 0.18	29.0	2.39 ± 0.34	3.11 ± 0.44	14.1
60 (1 h)	1.73 ± 0.46	0.65 ± 0.17	26.3	2.48 ± 0.35	3.23 ± 0.46	14.2
90 (1.5 h)	1.81 ± 0.44	0.68 ± 0.16	24.1	2.57 ± 0.37	3.35 ± 0.48	14.2
120 (2 h)	1.87 ± 0.45	0.70 ± 0.17	24.0	2.67 ± 0.35	3.47 ± 0.46	13.3
180 (3 h)	1.94 ± 0.44	0.73 ± 0.16	22.5	2.75 ± 0.34	3.58 ± 0.44	12.3
720 (12 h)	2.01 ± 0.42	0.75 ± 0.16	21.1	2.84 ± 0.32	3.69 ± 0.42	11.4
1440 (1 day)	2.05 ± 0.40	0.77 ± 0.15	19.6	2.90 ± 0.31	3.77 ± 0.40	10.7
4320 (3 days)	2.10 ± 0.37	0.79 ± 0.14	17.5	2.98 ± 0.30	3.87 ± 0.38	9.9
5760 (4 days)	2.15 ± 0.33	0.81 ± 0.12	15.2	3.05 ± 0.29	3.97 ± 0.37	9.4
10080 (7 days)	2.20 ± 0.29	0.83 ± 0.11	13.2	3.12 ± 0.28	4.06 ± 0.37	9.1
14400 (10 days)	2.27 ± 0.28	0.85 ± 0.11	12.4	3.18 ± 0.27	4.13 ± 0.35	8.5
20160 (14 days)	2.34 ± 0.27	0.88 ± 0.10	11.6	3.26 ± 0.26	4.23 ± 0.34	8.0
30240 (21 days)	2.44 ± 0.26	0.91 ± 0.10	10.8	3.33 ± 0.25	4.33 ± 0.32	7.4
40320 (28 days)	2.59 ± 0.26	0.97 ± 0.10	10.2	3.53 ± 0.12	4.59 ± 0.16	3.5
60480 (42 days)	2.88 ± 0.26	1.08 ± 0.10	9.1	3.67 ± 0.17	4.77 ± 0.22	4.7
76320 (53 days)	3.18 ± 0.26	1.19 ± 0.10	8.3	3.83 ± 0.18	4.98 ± 0.23	4.6
90720 (63 days)	3.25 ± 0.28	1.22 ± 0.10	8.5	3.99 ± 0.16	5.19 ± 0.21	4.1

[1]SD – 3 independently prepared material samples, analyzed 3 times; [2]CV – variation coefficient
